# A qualitative study of a new metric for estimating early-onset colorectal cancer risk in male veterans: “Colon Age”

**DOI:** 10.1186/s12875-025-02854-6

**Published:** 2025-07-15

**Authors:** Thomas F. Imperiale, Michael Cheng, Melissa R. Thomas, Marianne S. Matthias

**Affiliations:** 1 Center for Health Information and Communication, U.S. Department of Veterans Affairs, , Veterans Health Administration, Health Services Research and Development Service CIN 13 416, Richard L. Roudebush VA Medical Center, Indianapolis, IN USA; 2 Department of Medicine, Indiana University School of Medicine, Indianapolis, IN USA; 3 Center for Health Services Research, Regenstrief Institute, Indianapolis, IN USA; 4 Richard M Fairbanks School of Public Health, Indiana University Indianapolis, Indianapolis, USA

**Keywords:** Colon cancer, Risk prediction, Qualitative, Veterans health, Colonoscopy, FIT

## Abstract

**Background:**

In several Western nations, cancers of the colon and rectum have been steadily increasing in persons younger than age 50. Although the age at which to begin colorectal cancer (CRC) screening in the U.S. was lowered to 45 years in 2018, uptake of screening in persons aged 45–49 has been slow. Based on risk factors for CRC prior to age 50 and population-based CRC prevalence data, we previously defined a new metric for estimating the risk of CRC prior to age 50 called “Colon Age”. The objective of this study was to obtain qualitative data on the acceptance, feasibility, and clinical utility of this metric from patients and primary care providers.

**Methods:**

With permission from their providers, we recruited a convenience sample of average-risk male patients 35–49 years of age during their primary care appointment. Primary care providers were recruited through email invitation. Following informed consent, two interviewers conducted semi-structured qualitative interviews with participants. Interviews were conducted until saturation was reached. Interviewers were not involved in the tool’s development. The audio-recorded interviews were transcribed, de-identified, and analyzed using the constant comparison method.

**Results:**

Thirty-one (23 male Veteran patients, 8 primary care providers) interviews were conducted. Patients (mean age 47 years, 100% male) expressed willingness to follow screening recommendations from their provider, although most were unaware of other screening options beyond colonoscopy. Overall, patients expressed acceptance of the Colon Age concept and tool, finding it easy to understand, helpful for staying informed of their health, and a way to empower themselves in their screening decisions. Providers (mean age 53 years; 50% female) also found the tool acceptable, commenting on its usefulness for starting screening conversations with patients and improving screening uptake. Providers questioned the tool’s time commitment, consistency with practice guidelines, and the process of tool development.

**Conclusions:**

In this age of precision medicine, the Colon Age tool—despite some limitations—appears to be useful to patients and providers in individualizing risk for CRC and may improve uptake of screening in persons younger than age 50.

**Supplementary Information:**

The online version contains supplementary material available at 10.1186/s12875-025-02854-6.

## Background

Although colorectal cancer (CRC) morbidity and mortality are declining in persons aged 50 years and older, the incidence of early-onset CRC (i.e., prior to age 50) is slowly but steadily increasing [1]. Within the next two decades, the U.S. is estimated to have the highest number of new CRC cases [2], with increases of 28% and 46% in colon and rectal cancer, respectively, for patients aged 35–49 [3, 4]. CRC is now the most common and second most common cause of visceral cancer deaths in men and women, respectively, younger than age 50 [1, 5]. Since 2018, U.S. practice guidelines have evolved to recommend starting average-risk CRC screening at age 45 rather than age 50 [6–8], yet it remains unclear whether low-, average-, or high-risk individuals account for the modest uptake of screening in this age group [9, 10].

CRC, especially early-onset CRC, remains a heterogenous disease to detect and treat [2, 11]. Although several risk factors for early-onset CRC have been identified [12, 13], their use in screening decisions has yet to be defined well enough for clinical practice. Early detection of early-onset CRC through screening or early diagnostic evaluation is likely the best strategy for reducing incidence and mortality from CRC [14]. However, awareness of the need for screening, as well as uptake, among persons of different risk profiles younger than age 50 is unknown [15]. Although several scoring systems exist that can stratify risk for advanced neoplasia (the combination of CRC and advanced, precancerous polyps) [16–20], a risk model for estimating risk of CRC in persons younger than age 50 has yet to be identified. In an age with multiple risk models for CRC screening, comprehensibility and usability are key features of a model’s ultimate clinical utility [21, 22]. As such, a model with a meaningful metric for expressing risk is required to gain clinical traction for CRC screening in persons younger than age 50 [23, 24].

In lieu of an organized national CRC screening program, the U.S. currently relies heavily on primary care providers (PCPs) to discuss screening during annual exams or unrelated healthcare visits, known as opportunistic screening [25]. Therefore, the perspectives of PCPs who recommend early-onset CRC screening are critical to understand the uptake of new guidelines among the U.S. population younger than age 50. Preliminary studies show discordant views between PCPs and patients regarding screening preferences and options for testing [26–28], as well as misaligned communication between them [29]. Despite the necessity of PCP recommendations, little data exist regarding PCPs’ decision-making to endorse early-onset CRC guidelines in individual patient encounters and their acceptance of CRC risk models [30]. In the current study, we interviewed male Veterans and their PCPs to assess the comprehensibility, acceptance, and potential clinical utility of a ‘Colon Age’ metric to address the issue of CRC screening.

## Methods

This study was approved by the Indiana University Institutional Review Board and the Research & Development Committee of the Richard L. Roudebush Veterans Affairs (VA) Medical Center in Indianapolis, Indiana. Study participants provided written informed consent prior to the recording of interviews and verbal consent at the start of interview recordings, allowing for the use of their de-identified data. We used qualitative methods to allow for detailed accounts of perceptions of the risk prediction tool and Colon Age concept. Individual interviews were conducted with patients and PCPs between June and September 2022.

### Colon Age risk prediction tool

The Colon Age tool is a risk prediction model derived and split-sample validated in male Veterans. In a previous case-control study of risk factors for early-onset sporadic CRC in male Veterans, [31], we derived and validated two risk prediction models (a 15-variable model and a 7-variable model) with the goal of assisting providers in determining when, whether, and how to screen patients younger than age 50. Combining either model with CRC occurrence rates from the Surveillance, Epidemiology and End Results (SEER) data provides a metric called “Colon Age,” which estimates the age of a patient’s colon based on their risk factor profile. The tool calculates Colon Age by multiplying the patient’s SEER rate, based on age and sex, by a summative relative risk to produce a population risk or revised SEER rate for the individual. The summative relative risk is determined using the following patient-specific risk factors (from the 7-variable model): age, current alcohol use, Charlson Comorbidity Index, service connection status (whether a medical condition resulted from military service, directly or indirectly), statin use, nonsteroidal anti-inflammatory drug (NSAID) use, and CRC history in a first or second-degree relative [32]. Further information regarding the Colon Age tool’s variable selection and validation, as well as risk thresholds and screening recommendations derived from the tool, for both 7- and 15-variable models is published elsewhere [31, 32].

### Participants

Potentially eligible Veteran patients were identified through medical record review of the Roudebush VA Medical Center primary care clinic schedules. Patients were eligible if they were 35-to-49 years of age and considered to be average-risk for CRC. Patients were excluded if they had inflammatory bowel disease, prior CRC screening (either colonoscopy or fecal immunochemical test [FIT]), or a family history of CRC in one first-degree relative younger than age 60 or two second-degree relatives regardless of age. We also excluded female patients, due to the risk model’s development based on male Veterans [31, 32].

After obtaining permission from their PCPs, we approached patients during a clinic visit to explain the study and solicit their interest in participating. To achieve concept saturation, it is recommended to have a sample size of 12–20 participants [33]. Recruitment occurred until saturation was reached among patients, with the intention to recruit an equal proportion of patients in the age groups of 35–39, 40–44, and 45–49 years (*n* = 8 patients per age group). Following an initial e-mail invitation, PCPs were recruited based on convenience sampling. Because the PCPs represented a homogenous sample, it is consistent with published recommendations for qualitative research to recruit between 5 and 8 participants in order to achieve saturation [33]. All participants received a $25 gift card at the conclusion of their interview.

### Interviews

After participants provided informed consent, personnel not involved in the development of the tool conducted face-to-face, semi-structured interviews [34]. The interview questions were created by the study authors based on our desire to understand patient and provider knowledge about CRC and CRC screening and to determine their understanding and acceptance of the Colon Age risk tool. Interview data were collected via audio recording and stored electronically in study-designated files on secure servers. Each interview lasted approximately 30 min. During the interview, study participants were asked about their experiences with CRC screening and their perceived utility of the risk prediction model following the interviewer’s explanation of the Colon Age tool. For patients, the interviewer asked for verbal permission to input patient-specific data and show them their Colon Age using the 7-variable model. For the providers, the interview included questions about how they present CRC screening to their patients, whether they discussed screening test options with patients, and the providers’ perceived utility of the Colon Age tool in daily practice. Each interview was transcribed and de-identified for analysis. The interview guide for both patients and providers is available in the supplemental file.

### Data analysis

Interviews were analyzed by two authors who were not involved in the development of the Colon Age tool. Analysts used the constant comparison method [35], which consists of two phases: open coding and focused coding. In the first phase, reviewers read and familiarized themselves with each interview transcript. During these readings, emergent codes were identified and refined through multiple readings and discussion, with discrepancies resolved by consensus. Once the code list was stable and agreed upon by all authors, it was used to guide the next phase of analysis: focused coding. In this phase, each analyst applied codes to all transcripts, after which a third author reviewed all coding to ensure consistency and agreement.

## Results

**Fig. 1:**
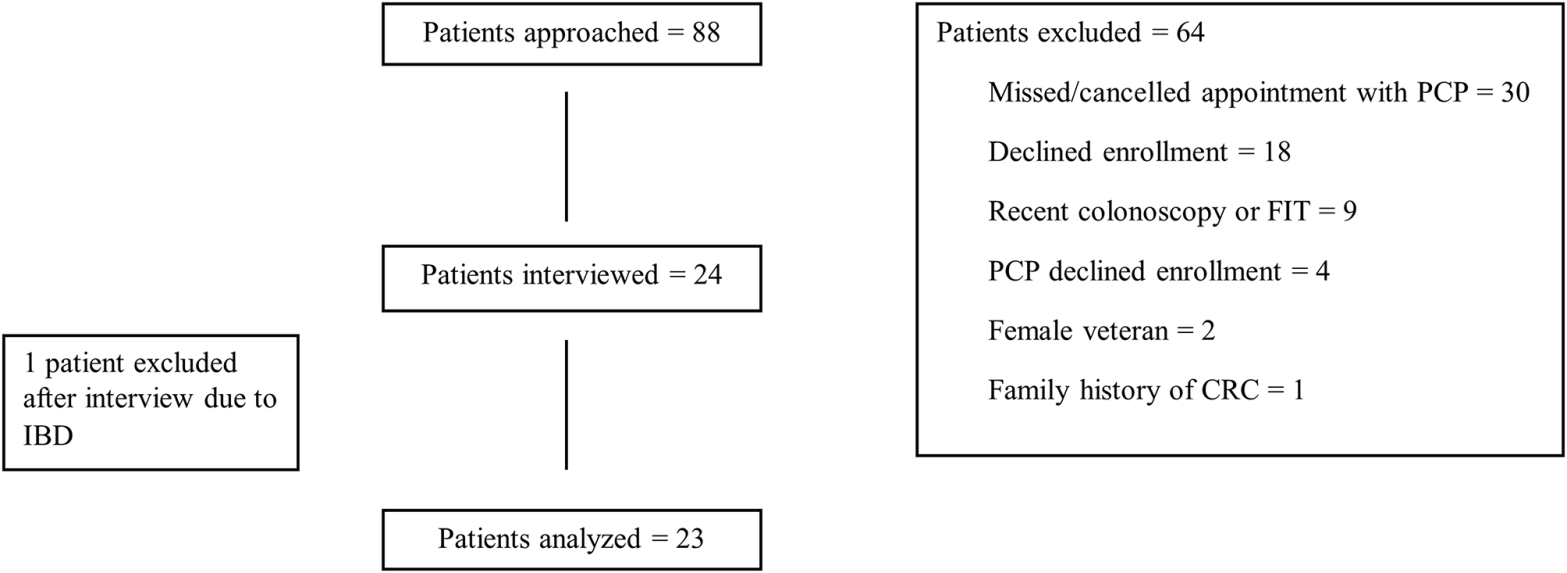
Flow chart of study participants (patients only)

Twenty-four patients and eight PCPs were recruited for this study (Fig. 1). Following his interview, one patient was excluded from analysis due to post-interview discovery of a diagnosis of Crohn’s disease, leaving 31 study participants (23 patients and 8 providers). Among the 23 patient participants, mean age was 47 (standard deviation [SD] = 4.9) years and 100% were male; among the 8 providers, mean age was 53 (SD = 9.8) years and 4 (50%) were female. The mean duration of medical practice was 29 years (range, 15–40 years), with a mean of 19 years of practicing at the VA (range, 5–27 years). All 8 providers were MDs or DOs.

### Patient perspectives

#### Patient experiences with screening

##### Awareness of screening

All patients were well-aware of CRC and acknowledged that they could develop it, but when asked about screening options, their knowledge varied (Table 1). Some patients had not heard of FIT, while all had heard of colonoscopy. Patients cited learning about testing options from their doctor, friends, or general media. While most patients had heard of FIT, some “*didn’t even know you could do the [FIT]*” or have “*never been advised by a doctor”* regarding their screening (PT7,22). After viewing the Colon Age tool, a patient mentioned never “*[going] through all of this [data]*” in conversation with a nurse practitioner: “*[she] just kind of told me there were two options*” (PT5).

**Table 1:** Overall patient themes for the Colon Age tool

Theme	Sub-theme	Supportive quotes (participant)
**Patient experiences with screening**		
1) Awareness of colon cancer screening	Aware of colon cancer risk in people younger than age 50	“People get cancers at very young ages…So, cancer doesn’t show prejudice when it comes to age.” (PT11) “Different lifestyle factors could make your organs older at a younger age, so not everybody is really knowledgeable of what they’re doing to themselves.” (PT16)
	Lack of knowledge regarding screening options	“I just wasn’t too informed on exactly how [screening] works.” (PT5) “I guess I don’t have any other source besides asking my doctor.” (PT16) “I really am not familiar with [colonoscopy].” (PT18) “I feel a bit weird about asking [for a colonoscopy] given my age, but I’m not opposed to asking.” (PT22)
2) Discussion with provider regarding screening preferences	Positive interactions with provider	“Most things, at least for my doctor if she recommended it, I would do it because I trust her.” (PT8) “For me, I just have to go with what is recommended by the medical community.” (PT2)
	Negative receptions of screening	“I didn’t even know you could do the [FIT].” (PT7) “When I had the conversation with the nurse practitioner [about screening], we didn’t go through all of this [data]. Just told me there was two options.” (PT5) “I’ve never been advised by a doctor or anything that I need to start getting [screening] done…A lot of this stuff I’m not familiar with.” (PT22)
3) Risk perception	Effect of age	“Being my age, it’s not something I really stressed or thought about. I figured that would [occur at] an older age.” (PT16) “I haven’t thought too much [about colonoscopy], as I know I’m getting closer to 40, and that was kind of on the list for when I hit 40.” (PT20) “I don’t think there’s much of a threat [of colorectal cancer] at the moment.” (PT1)
	Comfortable with not knowing whether cancer is present	“I don’t want to find out if I have cancer or not to be honest.” (PT8) “I don’t know if I would get tested for anything because I’m comfortable dying.” (PT23)
**Understanding and acceptability of the Colon Age tool**		
1) Usability of the tool	Acceptable for the patient	“If anything, I’d be completely okay filling this [tool] out, because the whole reason I want to start going to the VA is cause I want to take better care of myself.” (PT22) “I think if this tool is developed properly, a lot of people would do this to figure out if they need the FIT versus the colonoscopy.” (PT24) “I think [the tool] would definitely work out better for the veterans, or anybody really. Because it gives insight to what your body is actually doing.” (PT8)
	May help the doctor	“[The tool] provides detailed information to the doctor that could help make recommendations and helps a patient be informed about what is their best option to take [for screening].” (PT2) “I think the tool just helps the doctor to make a better decision on when to recommend the stool test or the colonoscopy…I think it’s very clear.” (PT17)
2) Tool is easy to understand	Easy to follow	“[The tool] was very easy to understand and comprehensive to follow along with.” (PT24) “[My Colon Age] makes me feel good.” (PT8)
	Helpful to understand your own risk for cancer	“I think [the tool] gives you a lot of information [about] what things you can do to help with your health, and see the long-term implications of drinking or such other factors, as like the aspirin and the ibuprofen.” (PT5) “I think that [the tool] would help people better understand their risk factors, but I think it’s simple to understand.” (PT25)
	Willing to calculate Colon Age	“I don’t play with my health or my family’s so if it’s something like [a colonoscopy], I’m going to take care of it.” (PT24)
**Barriers to use of the Colon Age Tool**		
1) Privacy and disclosure concerns	Unsure of the tool	“[The tool] seems like an extra step right now to me. That just gets in the way, like if my doctor knows my family history, he should have been on top of that. Like, I don’t see what the Colon Age does; that, to me, is just kind of a waste.” (PT15)
	Privacy of the data	“I would much more prefer to try and keep that [data for the tool] a little bit more private with my doctor’s team.” (PT20)
2) Comfort level with the tool	Understanding the ‘math’ behind the Colon Age	“I don’t understand the math behind [the tool], but I mean you plug in the numbers, and it calculates, it’s just like a calculator. I don’t need to understand where the numbers go and what they mean.” (PT11) “I have a question about the Colon Age. I guess, I don’t understand what the Colon Age is.” (PT8)
	Feeling uncomfortable with the tool	“Would I be comfortable with a doctor making medical decisions based on this tool alone? I would say not necessarily…I think it’s just a great tool to help drive what your decision is to go for treatment or diagnostics.” (PT2) “Some of [the tool] looks kind of foreign to me.” (PT16)

##### Screening preferences

Patients generally preferred FIT over colonoscopy. For one patient’s initial experience with being offered FIT by their provider, they mentioned “*I was actually glad to see that [FIT] was affordable*,* where you wouldn’t have to show up*,* less invasive*” (PT16). Another patient conceded that “*I don’t really want to have a colonoscopy unless I have to*” (PT6). Overall, patients showed great trust in their PCPs’ recommendations, often stating they would follow doctor’s orders even if they felt reluctant towards getting screened. One patient who believed FIT was less complete than a colonoscopy stated he would be comfortable with a FIT if the Colon Age tool was used to explain why: “*[The PCP] can say to me we don’t need to do [a colonoscopy]*,* you’re at a very low risk and it’s not something I would worry about at this point of time*” (PT9).

##### Risk perception

Overall, patients were aware that early detection of cancer was important for maintaining their health, but many believed that “*there’s [not] much of a threat”* until a later age. One stated: “*I haven’t thought too much into [screening]*,* as I know I’m getting closer to 40*,* and that was kind of on the list for when I hit 40*” (PT20). A few patients were comfortable with not knowing whether they had CRC, and therefore did not feel the need to get screened. One stated that he would forgo a screening test because a diagnosis of any form of cancer is “*a death sentence*” (PT10). While most patients mentioned feeling comfortable with death, all agreed that they wanted to either avoid cancer or catch cancer early, with one patient stating, “*I’m okay with death*,* but I want it to be on my terms*” (PT23).

### Understanding and acceptability of the Colon Age tool

#### Usability

Nearly all patients (96%) agreed to have their Colon Age calculated, with one patient declining due to privacy concerns. The majority (68%) of patients’ calculated Colon Age was below their biological age, while 14% of patients had a higher Colon Age, and 18% had a Colon Age equal to their biological age. Irrespective of their own Colon Age, patients saw benefits to using the tool for both patients and providers, with one stating: “*[The tool] provides detailed information to the doctor that could help make recommendations and helps a patient be informed about what is their best option to take [for screening]”* (PT2). Another patient saw benefits in the tool making “*a difference in your treatment [and] how fast [cancer is] being detected*” (PT19). Patients focused on the usability of the tool “*[assisting] the doctor*” to “*make a better decision*” (PT20,17). Additionally, a few mentioned that the tool would be good to use across the VA system.

#### Tool was easy to understand

Patients cited the simplicity of the tool as a large part of its acceptability. When first introduced to the tool, many found it “*foreign”* and asked questions, but found that “*once [the tool was] explained*,* you can understand it”* (PT12). Patients noted that the tool is “*very clear”* and “*easy to understand”* (PT17,24), particularly because it allows them to further consider their own health behaviors and how this may affect their Colon Age. For example, one patient mentioned that “*sometimes [it] gets confusing if [aspirin or ibuprofen] is helpful or not*” and that the tool helped them learn “*what things you can do to help with your health”* (PT5). In this sense, output of the Colon Age tool was helpful for patients, and the tool itself allowed them to feel a sense of involvement with their healthcare decisions.

### Barriers to use of the Colon Age tool

#### Comfort level

While the general consensus of the Colon Age tool among patients was decidedly positive, a single patient expressed skepticism about use of the tool for patients in his age group, stating the tool “*seems like an extra step right now for me*” and “*just [manipulates] a number*” (PT15). Although the tool was generally clear to most patients, a few seemed unsure of how the calculations worked, and this influenced their understanding of their own Colon Age. For example, one patient mentioned that “*I need to know what I’m looking at*,* being that I don’t understand the formula. I don’t know what these numbers mean…I don’t understand the math behind [the tool].*” (PT11). Another spoke on the limits of the Colon Age tool as a reliable method of decision-making: “*Would I be comfortable with a doctor making medical decisions based on this tool alone? Not necessarily…I think it’s just a great tool to help drive what your decision is to go for treatment or diagnostics [in screening]*” (PT2). This was supported by a patient who remarked that the tool has “*that missing piece of knowing my history and everything that is me*” (PT20) if screening discussions during encounter time are dedicated to filling out the tool.

#### Privacy and disclosure concerns

Patients were aware of the time pressures for their PCPs, and several stated that they may be able to provide the information for the tool on a pre-screening tablet in the waiting room. Some patients expressed a reluctance to fill out the tool themselves, due to crowding in the waiting room and privacy concerns, or due to questions about how certain factors would affect their Colon Age calculation, which cannot be answered without a healthcare member present. Another patient acknowledged the limitations of the tool when a patient is reluctant to share and receive information about how their lifestyle affects their health: “*Unfortunately*,* we know that some people are embarrassed…or don’t want to talk about their history and that could potentially be detrimental to getting an accurate age on the colon*” (PT20).

### Provider perspectives

#### Potential benefits of the Colon Age tool

##### Potential to increase uptake of screening

With a consensus among the medical community towards beginning CRC screening prior to age 50, PCPs agreed that CRC screenings were frequently discussed during their clinic sessions (Table 2). One provider stated: “*We are finding more younger and younger patients with colon cancer”* (PCP3). However, they noted that patients are less educated about testing options and less concerned about the threat of cancer: “*Some patients don’t want to know whether they have colon cancer or not and they refuse [the screening]*” (PCP1). PCPs discussed hesitation among patients to get screened prior to age 50 due to “old-fashioned” beliefs that “*everybody has to die [of something]*” (PCP8).

**Table 2:** Overall provider themes for the Colon Age tool

Theme	Sub-theme	Supportive quotes (participant)
**Potential benefits of the Colon Age tool**		
1) Use of risk model	Meaningful for decision-making	“I think it would give us another tool…If you have somebody that’s sitting on the fence between whether to get a FIT and a colonoscopy, if they know their Colon Age is younger than their stated age, maybe they would feel more comfortable getting a FIT.” (PCP5) “It’s a decision support tool. Like many other decision support tools, there is benefit in that.” (PCP4)
	Facilitate discussion between patients and PCPs about screening	“If [the tool] gets the patients involved and [is] increasing their knowledge, then it really has a chance to make a difference.” (PCP2) “[The tool] would start a conversation that might not have happened otherwise between the provider and the patient.” (PCP6)
	Has potential to increase uptake of screening	“I think [the tool] is a good idea because we are finding more younger and younger patients with colon cancer.” (PCP3) “We have seen some colon cancer earlier, so this might be helpful [to] offer them earlier screening.” (PCP8)
	Personalizes medicine to each patient	“The holy grail in medicine is finding the individual risk for certain events. Anytime you can get closer to somebody’s individual risk as opposed to just age cohort risk, it’s more personalized medicine.” (PCP6)
**Potential limitations of the Colon Age tool**		
1) Need for empirical evidence	Need for validation and support from professional societies	“I love the concept [of Colon Age] if it proves to be a better way to make the decision, meaning if it somehow correlates more closely with benefits and risks. I think it would have to be based on evidence.” (PCP2) “You need to validate this risk model before we would be comfortable in changing who we’re doing the colon cancer screening for.” (PCP5) “Well, if it actually is proven to benefit patients…[by] lowering the number of people who die from colon cancer, that’d be great! But if it’s not proven, I don’t know [if I’d use it].” (PCP7) “I like the idea of this tool if it is actually supported the profession and if it’s evidence based.” (PCP2)
	Patient reluctance towards screening	“There [are] cases where this may not be very clear whether they should be screened.” (PCP2) “Some patients don’t want to know whether they have colon cancer and they refuse [screening].” (PCP1) “The other concern is if somebody’s chronological age is 50 and the Colon Age comes to the calculation of 40, the person may not go in for the screening because the perceived risk may be lower than average.” (PCP4)
	Lack of education among patients	“Because not many patients will understand the Colon Age at all. They need more explanation.” (PCP1) “It’s your job as a provider to make sure that your patient is well-educated [about screening].” (PCP5)
2) Time pressure for providers	Implementation format	“I think if it could be integrated in the EHR, it would be much better.” (PCP3) “[Regarding] how to actually execute this or implement it…If it’s easy and it’s really streamlined and fast…then it’s pretty likely I would use it.” (PCP2) “I think this [tool] is great as long as the provider doesn’t have to make the calculations…It could be very time intensive otherwise, it seems.” (PCP6)
	Need for clinical reminders	“Usually, I just rely on the [colonoscopy] reminder [to discuss screening in appointments].” (PCP7) “[CPRS reminders] sound good in practice but, in reality, you get so much pop-up data that it becomes white noise. It’s a real challenge because there’s a lot of things that you need to do [for patients].” (PCP5) “We have a clinical reminder which is very helpful. So, it alerts us to discuss [screening] and I tell them all the importance of doing the screening…I make a determination depending on if they’re higher risk.” (PCP4)
	Interested in output more than process	“It should be integrated into the EHR…we just need a Colon Age. That’s what we are looking at. We just need one number: his Colon Age.” (PCP4) “I would love [seeing the Colon Age] with a screening recommendation.” (PCP6) “It would be much easier if there is a tool that says which [screening test] would be better, then give us the Colon Age.” (PCP1)

PCPs were supportive of the potential for the Colon Age tool to allow patients to consider their own risk factors, with the subsequent potential to increase their acceptance of CRC screening, especially among more reluctant patients. For example, one provider stated, “*The tool could be designed to help them actually understand what their own risk is and whether they should get this done…If it gets the patients involved and [is] increasing their knowledge*,* then it really has a chance to make a difference*” (PCP2). While most PCPs were hesitant to state how much screening would increase, they agreed that having patients complete the tool could get them engaged in screening decisions.

##### Potential to facilitate shared decision-making

All PCPs reported that they consider patients’ preferences when discussing screening options with their patients. When asked which test they recommend in clinic, the PCPs indicated they would first discuss risks and benefits of each test, and then allow the patient to make the decision. Shared decision-making was a common theme of discussing test options with patients, and all PCPs valued patient preferences for choice of a screening test: “*I recommend being screened for those who are eligible to be screened. I give them a choice of the two tests [FIT or colonoscopy]*,* and we talk through the advantages and disadvantages of each one*,* and then arrive at a decision from there*” (PCP2).

Not all cases are clear-cut, however. One PCP noted that sometimes, “*we have more of a difficult decision to make*,* I think*,* about whether that person should be screened. There are some gray zone cases*” (PCP2). Patients can often be unsure of which test they prefer between the colonoscopy and FIT, especially when they are considered to be average risk by their PCP. In these cases, PCPs indicated that the Colon Age tool would be useful for guiding a decision on whether to screen and which test to recommend. In particular, some PCPs invoked the increasingly important principle of precision medicine: “*The holy grail in medicine is finding the individual risk for certain events. Anytime you can get closer to somebody’s individual risk as opposed to just age cohort risk*,* it’s more personalized medicine*” (PCP6).

##### Potential to facilitate discussion between patients and PCPs

With concerns of time management in appointments across PCPs, many considered a benefit of the tool to allow greater patient autonomy regarding CRC screening. One provider thought that the tool *“would start a conversation that might not happen otherwise*,” (PCP6) and another agreed that the tool “*will create less dependence on the provider to bring up the issue [of CRC screening] because some may forget to do this otherwise*” (PCP2). The act of filling out the tool may allow patients to consider their own risk and start a discussion about CRC screening. This may allow the tool to facilitate personalized care and reduce the time it takes to have the conversation about screening test options during appointments.

### Potential limitations of the Colon Age tool

#### Need for empirical evidence

A concern among PCPs was the acceptance by the medical community in the application of the Colon Age tool. Many PCPs asked about the tool’s validation process, whether it was evidence-based, and whether there were any studies showing that the tool increased screening uptake. Without these supportive data, some PCPs stated that they would not use the tool: “*I don’t want [to] get into using something experimental on my own patients in my practice*,* so I want to know*,* is this method supported by professional societies*,* medical establishments*,* and my own institution?*” (PCP2). Some providers expressed skepticism that the tool would be useful for providing recommendations that would meaningfully affect clinical practice for patients who are in the “early” age group, with one provider stating, “*if somebody’s chronological age is 50 and the Colon Age comes to the calculation of 40*,* the person may not go in for the screening because the perceived risk may be lower than average*” (PCP4).

#### Time pressure for providers

All PCPs expressed a preference for the tool to be incorporated into the electronic medical record. Further, they would “*absolutely not*” (PCP7) use the tool in an external software app. One PCP stated: “*I cannot afford to spend 10 minutes on this when I have 30 minutes to address 20 other problems*” (PCP4). Another PCP stated that, for providers, “*time is essentially not available*” (PCP2). PCPs expressed interest in seeing the patient’s Colon Age, but all providers were against inputting the data into the tool themselves due to limited time in clinical visits. In other words, PCPs care about the result of the Colon Age tool, but do not want to be involved in the process of obtaining the data. One PCP stated: “*We just need one number: [the] Colon Age*.”

Some PCPs considered the benefit of allowing patients to fill out information directly to save PCPs time in the appointment, while still gathering the required data for the tool. Many PCPs rely on clinical reminders of CRC screening to bring up the issue of screening with their patients during the clinical encounter, and therefore they recommended that the Colon Age “*comes looped in with the [automatic] recommendation*” (PCP6). These recommendations for whether and how to screen would require the integration of the Colon Age tool within the institutional electronic medical record to automatically notify the provider of the patient’s Colon Age. Overall, PCPs agreed that they would only use the tool if it were “*time neutral*” and already automated into their current applications.

## Discussion

As early-onset CRC is rising at a slow, but steady, rate among persons younger than 50 years of age, the need exists to identify those at risk and improve adherence to CRC screening guidelines among this age group. Moreover, since half of early-onset CRC occurs in persons younger than age 45 [36], it may–at some point– be appropriate to extend screening to some patients who are younger than 45 years. Although estimating the risk of CRC remains imprecise, the Colon Age tool was designed to facilitate patient-provider discussions for screening options for these younger patients based on an estimate of individualized risk that is understandable to patients. Of the multiple risk models that have been developed for CRC, few have assessed understanding and acceptability from the perspective of both providers and patients [21].

In this study, patients and PCPs were presented with a recently developed risk prediction model and asked about their perspectives when making screening decisions or in clinical practice, respectively. Based on these interviews, patients were found to be generally accepting of the Colon Age tool, given adequate privacy measures are put in place, and providers tended to acknowledge the tool’s potential positive impact on patients’ screening rates, while sharing concerns regarding its validation and use of time. We found that patients had a poor understanding of screening alternatives to colonoscopy, such as FIT. Providers generally stated that they discussed screening test options with their patients, yet patient awareness of screening options for CRC was low in this small sample. These views from patients and PCPs revealed important benefits and barriers when considering use of the Colon Age tool among persons younger than age 50, including benefits such as (1) patient and clinical usability; (2) ease in understanding the tool; (3) potential to improve screening uptake; (4) potential of facilitating shared decision-making; and (5) potential to facilitate discussions about the need for CRC screening; and limitations such as (1) low comfort level among patients when initially presented the tool; (2) concerns among patients regarding data privacy; (3) provider uncertainty regarding alignment with clinical guidelines; (4) limited time for discussion during clinic visits; and (5) PCPs’ preference that the tool be integrated into the electronic medical record to facilitate its use. Regardless, patients in the current study were accepting of CRC screening and recognized how the Colon Age tool could benefit their personal health.

Both patients and providers agreed that the tool would be useful for determining whether an average-risk male patient should undergo a colonoscopy or FIT, as determined by the PCP and patient. The Colon Age tool provides arbitrary thresholds for decision-making about screening based on the revised SEER rate. For example, a 47-year-old male with no risk factors in the 7-variable model may have a “low-risk” Colon Age of 35–39 years [31], in which case non-invasive screening, or even deferring screening, is supported. An “intermediate” risk scenario would indicate at least non-invasive screening, such as annual FIT, in an instance where a 47-year-old male patient preferred non-invasive screening and had the Colon Age of a 45–49 year-old [31]. On the other hand, for a 47-year-old male with a “high-risk” Colon Age of 80–84 years, given that he is non-service connected, has a Charlson Comorbidity Index of 1, has family history of CRC, currently uses alcohol and does not use NSAID or statins, colonoscopy would be recommended [31].

In support of effective patient-provider communication, clinical outcomes including decision-making, adherence to treatment plans, and future healthcare utilization have been shown to improve when patients are involved in decisions about their care, such as determining an appropriate screening test for their own risk profile [37–39]. A few PCPs in this study highlighted the clinical benefits of precision medicine when using individualized risk assessment models such as the Colon Age tool. Given the case where males are experiencing disproportionally higher rates of early-onset CRC, yet have lower rates of screening uptake than females [40], tools that invoke principles of patient-centered care, such as individual patient risk or shared decision-making [24], may contribute to improved self-efficacy of CRC screening among male patients.

The Colon Age tool is unique in its output of individualized risk, expressing risk in terms of estimated age of the person’s colon, which can drive a discussion about the need for a certain type of CRC screening. Although many CRC risk scoring systems exist, we have a limited understanding of the utility and acceptability of these scoring systems in practice. In a recent review of CRC risk prediction models, only 20% and 12% of models showed high potential clinical usability and clinical utility, respectively [21]. Other risk assessment tools for CRC as well as breast cancer, a similar disease with rising numbers of incidence cases in younger persons [41], are based on molecular and genetic considerations, which may be less feasible for wide-scale prevention across patient populations.

Enhancing our understanding of patient preferences for screening discussions and providers’ decision-making processes for screening recommendations complements preliminary findings of patient-based factors for screening uptake [28, 30], and may help to improve screening rates in the future. For the Colon Age tool, PCPs and patients shared similar perceptions about the tool’s potential benefits, but held discordant views regarding the accuracy of the tool relative to its simplicity for patient understanding. Where composite advanced risk models may show greater impact on screening rates [42], they may not be as easy to understand as simple models, such as the Colon Age tool. Finding a balance between patient usability and effectiveness of the tool is critical when considering subsequent studies of feasibility for early-onset CRC risk prediction models [21].

PCPs shared limitations of the Colon Age tool, largely regarding time constraints during patient appointments. Where patients saw benefit in learning about their personal health and discussing different risk factors while inputting their health history into the tool, PCPs were reluctant to complete the tool during their patient encounter time and preferred that the Colon Age be automatically calculated and integrated with clinical reminders. While PCPs were clear that they would not use the tool if not incorporated into their institutional electronic medical record system, recent studies have shown the impact of “alert fatigue” among physicians, particularly those in primary care who are expected to address prevention in addition to ongoing conditions with their patients [43, 44]. Alert fatigue occurs when there are large numbers of clinical reminders for providers, many of which may ultimately be ignored. To implement the Colon Age tool in practice, refinements in deployment and use may be considered with these provider and patient perspectives in mind.

### Strengths and limitations

This study is limited in that it was conducted at a single VA Medical Center in the Midwest, and all patients who participated were male. Accordingly, results may not apply to non-Veteran patients, including female patients, as well as non-VA providers. Further, there are limitations with the Colon Age tool itself, which was based on a risk prediction model having modest discrimination, and whose linkage to whether and how to screen were based on arbitrary SEER thresholds chosen by the developers of the Colon Age tool [31].

This study has strengths worth noting. The interviews and data analysis were conducted by members of the research team who were not involved in development of the Colon Age tool, reducing the potential for bias. The qualitative data, with its rich details of individuals’ perspectives and experiences, provides an in-depth picture of the Colon Age tool unable to be obtained from other methods. As such, the detailed and specific responses of participants provide important guidance for future modification and implementation of the tool. Thus, this study offers unique insights into patient and providers’ perspectives of the Colon Age risk prediction model.

## Conclusions

As early-onset cases of CRC continue to rise, a risk prediction model like the Colon Age tool, acceptable to both patients and providers, could positively impact patient perceptions and attitudes towards CRC screening. Providers and patients in this study reached a consensus that the Colon Age tool could improve uptake of CRC screening through individualized risk assessment and decision-making needed in the primary care setting.

## Electronic supplementary material

Below is the link to the electronic supplementary material.

Supplementary Material 1

## Data Availability

The datasets generated and analysed during the current study are not publicly available due to the private nature of the recorded interviews, but are available from the corresponding author on reasonable request.

## Abbreviations

CRC: Colorectal cancer
PCP: Primary care provider
VA: Veterans Affairs
SEER: Surveillance, Epidemiology and End Results
NSAID: Nonsteroidal anti–inflammatory drug
FIT: Fecal immunochemical test
