# Supplementary material for: A qualitative study of a new metric for estimating early-onset colorectal cancer risk in male veterans: “Colon Age”
Source: BMC Prim Care. 2025 Jul 15;26:226. doi: 10.1186/s12875-025-02854-6 (PMC12265148; doi:10.1186/s12875-025-02854-6)
Supplement: Supplementary file 1 — Supplementary Material 1 [file 12875_2025_2854_MOESM1_ESM.docx]

**Suppl Table 1. Patient Interview Guide***

**_____________________________________________________________________**

**History**

- Have you ever been tested for colon cancer?
  - If yes: when? Why did you get tested? (If they say because their doc recommended, ask: Were there any other reasons?)
    - What kind of test was it (e.g., colonoscopy, stool test, etc.)?
  - If no: Why not? (Probes: time, not wanting to go through a colonoscopy)
    - What would make you get tested? (probes: close friend or relative with CRC, fear, “because I’m supposed to”)

**Physician-Patient Communication**

- Have you and your doctor talked about having a test for colon cancer? (If this is already known from above questions, and they have discussed, ask: How have you and your doctor talked about having a test for colon cancer?)
  - If yes: How did that conversation go? (Probes: Has he/she brought it up recently? If so, what did you say?)
  - Did your doctor talk with you about the different ways you can get tested for colon cancer? What did he/she say? What was your reaction?
- (Ask if not covered above) If your doctor recommended a colonoscopy, what would you do? Explain.
- (Ask if not covered above) If your doctor recommended a test checking for blood in your stool, instead of a colonoscopy, would you be OK with that? (Explain).

**Knowledge/Barriers**

- Do you plan to have a (or another) colon cancer test any time soon?
  - If no, what is the biggest reason why you haven’t been tested? What would change your mind?
  - If yes, when? Is it scheduled?
- What do you know about how colon cancer screening is done?
  - Where do you get your information from?
  - (If they only talk about colonoscopy): Did you know there’s at least one other test available? (RA: explain that it’s a test for blood in the stool. It is recommended every year, which is different from colonoscopy, which is every 10 years. Both tests are about equal for detecting cancer.)
  - Did you know this before? Does this information change how you think about getting tested for colon cancer? Why (not)?

**Risk Tool**

- Intro: There is a new aide available to help estimate your risk of having colon cancer or precancerous polyps (most of the risk is for the polyps). For patients at very low or low risk of colon cancer or precancerous polyps, a test that is simpler than colonoscopy could be recommended. For patients at high risk, colonoscopy would be recommended.
  - What is your opinion of a colon age tool being used to recommend colon cancer screening?
  - Do you believe being screened for colon cancer at age 45 is too young, old or appropriate?
- Take patient through the slides.
- Take patient through the colon age tool
  - Ask the patient about all factors on the tool
  - Show patient CCI and ask about recent diagnoses, medications taken outside of VA, and frequency of smoking/alcohol use.
- How easy is it to understand this tool?
  - If not easy: what could make it easier to understand?
  - What could be clearer?
  - Ask patient if he knows what will happen to his colon age if his individual risk factors change (comorbidities, medication use, alcohol consumption)
  - Ask patient if he feels comfortable answering these questions either with a medical assistant, nurse, or pre-screening tablet.
- How would you feel if your doctor used this tool to help find the best colon cancer test for you?
  - If you were “low risk” and your doctor recommended a stool test every year instead of colonoscopy, would that be okay? (Probes: Would you be comfortable with a stool test? Would you be relieved that you wouldn’t need a colonoscopy?)
  - What is your opinion of a colon age tool being used to recommend colon cancer screening?
  - Do you believe being screened for colon cancer at age 45 is too young, old or appropriate?
- Is there anything else about this tool or about colon cancer testing that you’d like to share before we end the interview?

**______________________________________________________________________**

* Modified from Matthias MS & Imperiale TF. *BMC Family Practice* 21:43, 2020.

**Suppl Table 2. Physician Interview Guide ***

**Decision Making/Pt-provider Communication**

- How do you bring up the topic of CRC screening with your patients? What do you typically tell them?
  - What options if any do you present for screening? Why do you present different options (or why not)? Does this vary based on the patient? If so, how?
  - If a patient refuses colonoscopy, do you offer other screening tests? (if no, why not) (If yes) How do patients usually respond when you offer alternative tests? (Probe for specifics if necessary)
  - Do you discuss risks of screening? (If no, why not?) What do you say about risks?
- When you choose a screening test for your patients, do you tend to recommend the same test for everyone?
  - Do you take a patient’s individual risk into consideration? How likely are you to ask the patient for his/her preference and use this to inform your recommendations?
    - How is the final decision made (i.e., do you involve the patient in the decision making?)?
  - (Ask if not covered above) Has COVID-19 affected how you screen?
- When patients choose not to be screened for CRC what are their reasons? How do you address these?
  - (Ask if not covered above) What are the most significant barriers that may prevent patients from getting screened?

**Use of the Risk Model**

- (Assuming an average-risk patient with no specific screening preferences) What do you think about this risk index?
  If this tool were integrated into the Electronic Health Record, how likely would you be to use it to discuss CRC screening with your patients?
  - (if likely) What about the tool appeals to you?
  - (if not likely) What makes you unlikely to use this tool? (would need to probe into responses)
- What do you think about having a nurse or medical assistant input the data for patients and provide you with the results?
  - What do you think about having the patient input this data themself on a screening tablet in the waiting room?
- If this tool were developed into a software app, would you use it? If no, why not?
- Which of the two modalities – a software app or integration into the electronic medical record - has greater appeal to you? (If EHR): How would you like to see this tool integrated into the EHR?
- Do you think a tool like this would increase uptake of CRC screening by patients who are not up to date with their CRC screening? Why or why not?
- Could using a tool like this help you in your practice? If so, how? (probes: aids clinical judgment, provides understandable risk estimate, saves time) If NOT, why not?
- What are the disadvantages of a tool like this? (Ask the following probes after the interviewee’s open response: Are you concerned about: missing a cancer, getting sued, inadequate validation of the tool, not trusting the model, the tool taking away your clinical judgment, too time-consuming to use)
- Is there anything else about this tool or about colon cancer testing that you’d like to share before we end the interview?

***______________________________________________________________________***

* Modified from Matthias MS & Imperiale TF. *BMC Family Practice* 21:43, 2020.
